# The Impact of Diet on Urinary Risk Factors for Cystine Stone Formation

**DOI:** 10.3390/nu13020528

**Published:** 2021-02-06

**Authors:** Roswitha Siener, Norman Bitterlich, Hubert Birwé, Albrecht Hesse

**Affiliations:** 1 Department of Urology, University Stone Center, University Hospital Bonn, 53127 Bonn, Germany; hubert.birwe@hebgmbh.de (H.B.); albrecht-hesse@web.de (A.H.); 2 Department of Biostatistics, Medicine and Service Ltd., 09117 Chemnitz, Germany; bitterlich@medizinservice-sachsen.de

**Keywords:** dietary treatment, dietary protein, sodium chloride, methionine, phosphorus, urinary pH, urinary phosphate excretion, urinary cystine excretion, cystinuria, urolithiasis

## Abstract

Despite the importance of dietary management of cystinuria, data on the contribution of diet to urinary risk factors for cystine stone formation are limited. Studies on the physiological effects of diet on urinary cystine and cysteine excretion are lacking. Accordingly, 10 healthy men received three standardized diets for a period of five days each and collected daily 24 h urine. The Western-type diet (WD; 95 g/day protein) corresponded to usual dietary habits, whereas the mixed diet (MD; 65 g/day protein) and lacto-ovo-vegetarian diet (VD; 65 g/day protein) were calculated according to dietary reference intakes. With intake of the VD, urinary cystine and cysteine excretion decreased by 22 and 15%, respectively, compared to the WD, although the differences were not statistically significant. Urine pH was significantly highest on the VD. Regression analysis showed that urinary phosphate was significantly associated with cystine excretion, while urinary sulfate was a predictor of cysteine excretion. Neither urinary cystine nor cysteine excretion was affected by dietary sodium intake. A lacto-ovo-vegetarian diet is particularly suitable for the dietary treatment of cystinuria, since the additional alkali load may reduce the amount of required alkalizing agents.

## 1. Introduction

Cystinuria accounts for less than 1% of urinary stones in adults but up to 8% of urolithiasis in children and adolescents [1,2]. Cystinuria is a genetic disorder of the proximal tubular reabsorption of cystine and the dibasic amino acids arginine, ornithine, and lysine [3,4,5,6]. The hyperexcretion and poor solubility of cystine in urine can lead to crystallization and a lifetime risk of recurrent stone formation. The subsequent need for multiple stone removal procedures may contribute to impaired renal function [7]. Cystinuria requires consistent lifelong therapy. For patients who fail to respond to dietary measures and alkali therapy, pharmacotherapy with the currently available chelating thiol drugs, alpha-mercaptopropionyl glycine and D-penicillamine, is indicated. However, a variety of side effects, such as alterations in taste perception, mucocutaneous lesions, immune-mediated diseases, hematological reactions, proteinuria, and nephrotic syndrome, may necessitate discontinuation of medical therapy [8].

Dietary treatment plays a pivotal role in the management of cystine stone formation. The primary treatment strategy for cystine stone disease is focused on reducing 24 h urinary cystine excretion and concentration and increasing urinary cystine solubility [9,10,11]. High fluid intake to decrease urinary cystine concentration is the most important measure to prevent recurrences in patients with cystinuria. Furthermore, restricting dietary protein intake is recommended for two reasons. First, reducing the dietary intake of cystine and methionine is considered to decrease urinary cystine excretion [12]. Second, ingesting a high-protein diet with small amounts of plant foods may reduce urine pH and counteract alkalinization therapy due to the high content of the sulfur-containing amino acid methionine [13,14]. In addition, restricting dietary sodium to less than 2.3 g/day is recommended for patients with cystinuria based on the assumption that reduced intake of dietary sodium will lower urinary cystine excretion [15,16,17]. However, neither of these dietary measures has been demonstrated to reduce cystine stone formation in clinical trials.

A usual Western diet may promote cystine stone formation, as methionine is contained in substantial quantities in meat, sausages, fish, eggs, and dairy products [9]. Cystine stone patients are therefore advised to maintain a balanced mixed diet with moderate animal protein content [9,18]. A vegetarian diet theoretically may further reduce urinary cystine excretion and increase cystine solubility by increasing urine pH. However, there has been little investigation on the contribution of dietary modifications to the urinary risk profile of cystine stone formation. Studies on the physiological effects of diet on urinary cystine and cysteine excretion are lacking. The aim of this trial was to evaluate the impact of a Western-type, a balanced mixed, and a lacto-ovo-vegetarian diet on urinary risk factors for cystine stone formation in healthy subjects under controlled standardized conditions.

## 2. Materials and Methods

### 2.1. Study Design

Ten healthy men were enrolled in this study. Each participant had normal findings on clinical examination and Combur-9 test (Roche Diagnostics). The individuals took no dietary supplements or medications during the study. The subjects received three diets for five days each. The standardized diets were a Western-type diet (WD), a balanced mixed diet (MD), and a balanced lacto-ovo-vegetarian diet (VD). The composition of the diets is presented in Table 1.

The WD corresponded to usual eating patterns. Typical for a Western diet is a high intake of protein, methionine, cystine, and phosphorus. In contrast, the composition of the balanced MD and VD corresponded to the dietary reference intakes of the German, Austrian, and Swiss Societies of Nutrition [19]. The MD and VD contained similar amounts of the main nutrients fat, carbohydrates, and total protein. The daily fluid intake through beverages was 1.5 L on the WD and 2.5 L on the MD and VD. Dietary sodium intake was assessed by urinary excretion [20,21,22]. The composition of each standardized diet was estimated using PRODI version 5.3 (WVG, Stuttgart, Germany). The standardized dietary regimen, i.e., consistent daily intake of prescribed foods and fluids, leads to a steady state of metabolism so that consistent urinary values are achieved [23]. Daily 24 h urine samples were collected and analyzed for urinary parameters to ensure adjustment to each diet. The Ethics Committee of the Medical Faculty of the University of Bonn (01889) approved the study and informed consent was obtained.

### 2.2. Analytical Procedures

Urine volume, density, pH (potentiometry), urinary sodium, calcium and potassium (flame emission spectrophotometry), chloride (coulomb metric titration), ammonium (ion selective electrode), inorganic phosphate (phosphate molybdate reaction), inorganic sulfate (nephelometry), and creatinine (Jaffé reaction) were analyzed. Laboratory quality certification was available for each parameter. Urinary cysteine and cystine were determined using reversed-phase high-performance liquid chromatography (HPLC) with pre-column derivatization of amino acids [24]. The ion activity product of cystine was determined as described by Tiselius [25].

### 2.3. Statistical Analysis

Nonparametric Friedman one-way repeated measure analysis of variance (ANOVA) by ranks was used to explore the effects of different diets on urinary parameters. The Holm–Bonferroni method was used to adjust for multiple pairwise testing of diets. Comparisons of outcome variables by diet were completed with generalized linear models with an identity link and a per-person random effect. Spearman’s rank correlation coefficient (R) was applied to assess the direction and strength of the linear relationship between two variables of each diet. The last day of each diet served as control, since steady-state conditions were then attained. The significance level was considered as *p* < 0.05. All statistical tests were two-sided. Statistical analysis was performed using SPSS^®^ for Windows version 25.0 (IBM, Armonk, New York, NY, USA).

## 3. Results

A total of 10 men aged 21–32 years (mean ± SD: 27.7 ± 3.1 years) were included in the study and completed all visits. The mean body height, weight, and body mass index (BMI) of the participants was 178.7 ± 4.5 cm, 78.9 ± 11.7 kg, and 24.7 ± 3.7 kg/m^2^, respectively.

Table 2 presents the urinary risk profile on the last day of the WD, MD, and VD. Both urinary cystine and cysteine excretion tended to be lower on the VD compared to the WD, although the differences were not statistically significant. Urinary cystine excretion was 131, 97, and 103 µmol/24 h on the WD, MD, and VD, respectively. The urinary cystine-to-cysteine ratio did not change throughout the study. Urinary cystine concentration was significantly lower on the MD and VD compared to the WD due to significant differences in urinary volume, which is also reflected by urine density. 

Urine pH was significantly lowest on the WD and highest on the VD. Urine pH was at the upper physiological limit of 6.8 on the lacto-ovo-vegetarian diet. Urinary ammonium excretion was inversely related to urinary pH and potassium excretion on each diet. Urinary sodium and chloride excretion differed significantly between diets and were highest on the WD, moderate on the MD, and lowest on the VD. Urinary sulfate excretion was significantly lower on the MD and VD than the WD. Urinary phosphate excretion was significantly higher on the WD compared to the VD. Urinary calcium excretion was significantly highest on the WD and lowest on the VD. Urinary creatinine excretion did not differ between the study phases. The ion activity product of cystine was significantly lower on the MD and VD than on the WD.

**Figure 1:**
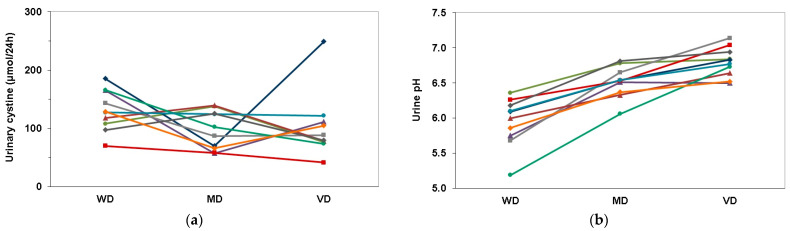
Individual course of 24 h urinary parameters of all participants on the different diets: (**a**) Urinary cystine excretion; (**b**) Urine pH. Abbreviations: WD, Western-type diet; MD, balanced mixed diet; VD, balanced lacto-ovo-vegetarian diet.

Figure 1a,b shows the individual course of 24 h urinary parameters of participants on the different diets. Compared to the WD, the VD resulted in decreased urinary cystine excretion in 9 of the 10 individuals (Figure 1a). In all 10 participants, urine pH was highest on the VD (Figure 1b). A sensitivity analysis without the outlier confirmed the results.

**Figure 2:**
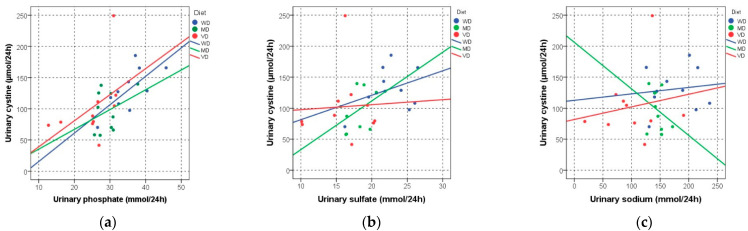
Association of 24 h urinary cystine excretion and suggested urinary risk factors for cystine stone formation (**a**) Urinary phosphate excretion; (**b**) Urinary sulfate excretion; (**c**) Urinary sodium excretion. Abbreviations: WD, Western-type diet; MD, balanced mixed diet; VD, balanced lacto-ovo-vegetarian diet.

Figure 2a–c demonstrates the association of 24 h urinary cystine excretion and suggested risk factors for cystine stone formation. Urinary phosphate was positively correlated with urinary cystine excretion on the WD and VD (WD: R = 0.709, *p* = 0.022; MD: R = 0.261, *p* = 0.467; VD: R = 0.648, *p* = 0.043) (Figure 2a), while urinary cystine excretion was neither associated with urinary sulfate (WD: R = 0.139, *p* = 0.701; MD: R = 0.418, *p* = 0.229; VD: R = −0.006, *p* = 0.987) (Figure 2b) nor with urinary sodium excretion (WD: R = −0.006, *p* = 0.987; MD: R = −0.188, *p* = 0.603; VD: R = 0.176, *p* = 0.627) (Figure 2c).

Additional analysis revealed that urinary calcium excretion was neither correlated with urinary cystine (WD: R = 0.588, *p* = 0.074; MD: R = −0.248, *p* = 0.489; VD: R = 0.345, *p* = 0.328) nor with urinary phosphate excretion (WD: R = 0.212, *p* = 0.556; MD: R = −0.297, *p* = 0.405; VD: R = 0.418, *p* = 0.229). Moreover, urinary cystine excretion was not associated with urine pH on the MD and VD (WD: R = −0.745, *p* = 0.013; MD: R = 0.231, *p* = 0.521; VD: R = −0.309, *p* = 0.385).

Univariate regression analysis confirmed that neither urinary sodium nor sulfate excretion was a predictor of urinary cystine excretion, but phosphate excretion was (Table 3).

In addition, univariate regression analysis revealed that neither urinary sodium nor phosphate excretion was a predictor of urinary cysteine excretion, but sulfate excretion was (Table 4).

## 4. Discussion

The dietary management of cystine stone formation comprises high fluid intake, urine alkalinization, and attempts reducing urinary cystine excretion by limiting protein and sodium intake. However, studies on the contribution of diet to the urinary risk profile for cystine stone formation are limited. In the present study in healthy subjects, 24 h cystine excretion was highest with intake of the Western-type diet (WD). The dietary protein restriction of 32% on the balanced MD and VD led to a reduction in urinary cystine excretion of 26% (WD vs. MD) and 22% (WD vs. VD). Due to the high variability of urinary cystine excretion, the differences in this parameter were not statistically significant.

A study conducted by Rodman et al. [12] of seven patients with cystinuria aged 17 to 35 years reported a significant decline in the mean urinary excretion of 1/2-cystine by 20% from 6.13 mmol/24 h on a high-protein diet to 4.89 mmol/24 h on a low-protein diet. The mean total protein intake on the low-protein diet was 54 g/day with 27 g from plant sources, while the protein-rich diet contained a minimum of 140 g/day of total protein with only 40 g from plant sources. However, the diets chosen for that study were relatively extreme. The protein content of the protein-rich diet was higher than what the majority of people customarily eat, and with the low-protein diet, it was difficult to meet the recommended dietary requirements for most adults [12]. A previous study of a 7-year-old girl with cystinuria found that severe restriction of protein to 20 g per day compared with 117 g/day reduced urinary cystine excretion by 31% [26]. 

A diet low in cystine and its precursor methionine is assumed to diminish urinary cystine excretion. The essential amino acid methionine is abundant in animal protein with high biological value such as meat, sausages, fish, eggs, and cheese. In comparison to animal protein, plant-based protein generally has lower cystine and methionine content. Surprisingly, in the present study of healthy subjects, 24 h sulfate excretion did not predict the outcome of urinary cystine excretion. Urinary sulfate excretion is considered as a biomarker of sulfur-containing amino acids, as sulfate is generated by the metabolism of methionine and cysteine [14,21]. From the present findings, it is concluded that the amount of dietary sulfur-containing amino acids was not the major determinant of urinary cystine excretion.

Univariate regression analysis revealed that urinary phosphate, instead of sulfate excretion, was a predictor of urinary cystine excretion. Under physiological conditions, 24 h urinary phosphate excretion has been regarded as a reliable marker of net gastrointestinal phosphate absorption, which in turn is related to the type and quantity of phosphorus present in the diet [27]. Morimoto et al. [28] reported that 24 h urinary phosphate excretion can be applied to estimate the amount of dietary phosphorus intake in healthy subjects, and that it may even be superior to estimates using weighed dietary records. Trautvetter et al. [29] confirmed that 24 h urine collection should be used to assess dietary phosphorus intake. Although the dietary phosphorus content of the VD was between the other diets, urinary phosphate excretion was significantly lower only on the VD compared to the WD. Phosphorus from organic phosphates is part of the animal and plant protein present in meat, sausages, milk and milk products, bread, and nuts. While phosphorus from plant sources is less digestible and hence less available for absorption than phosphorus from animal sources, processed foods containing inorganic phosphates as additives have the maximum potential bioavailability [27,30]. Unfortunately, we were not able to identify the amount of added inorganic phosphorus in the different diets; however, it is suggested that processed foods included in the Western-type diet, such as sausages and soft drinks, contain highly bioavailable phosphate additives [31]. A recent study on the effect of thiol drugs on patients with cystinuria demonstrated that 24 h urinary phosphate excretion was a predictor of cystine-binding capacity [32]. Whereas in a non-enhanced mixed diet, the digestible phosphorus is closely correlated with total protein content [30], the exact mechanism by which dietary phosphorus intake is associated with urinary cystine excretion is not clear. 

In this study of healthy subjects, 24 h urinary sulfate excretion was a predictor of urinary cysteine excretion. In food protein, cysteine exists mainly in the form of cystine because cysteine is rapidly oxidized to cystine in normoxic conditions [33]. Cysteine can usually be synthesized in the body under normal physiological conditions if a sufficient quantity of methionine is available. The significant association of urinary sulfate excretion with urinary cysteine excretion suggests that urinary cysteine is derived mainly from dietary methionine and cystine. In urine, cystine and cysteine are in a redox state [24]. Reductive substances cleave the disulfide bond of cystine, leading to the conversion of cystine to its more soluble monomer cysteine. For monitoring of therapy, the differentiation between cystine and cysteine is essential, and it can only be achieved by using high-performance liquid chromatography (HPLC)-based analysis [24]. In the present study, no significant differences in the cystine-to-cysteine ratio were observed between the diets. 

Since diets low in protein have demonstrated no clear benefit and can lead to diminished intake of essential amino acids, total protein intake should not be restricted below the dietary reference value of 0.8–1.0 g protein per kg ideal body weight per day for adults. In the present study, the total protein content of the balanced mixed diet and the lacto-ovo-vegetarian diet of 0.8 g per kg body weight per day meets the recommended intake and is intended for long-term use.

Restricting dietary sodium intake has been suggested to be an effective measure to reduce urinary cystine excretion in patients with cystinuria [15,16,17,34,35]. A study of four patients with cystinuria reported that urinary cystine excretion can be expected to decrease by about 650 µmol per 24 h if dietary sodium intake is reduced by 150 mmol per day [15]. Norman and Manette [16] confirmed the findings in five adult patients with recurrent cystine stone formation. Restricting dietary sodium intake from 4200 to 2000 mg/day resulted in a significant decline in mean cystine excretion by 51%. In five children with cystinuria, Rodriguez et al. [35] showed that a low-salt diet was effective in reducing urinary cystine concentration. A study of 79 samples of 24 h urine from 69 patients with cystine stones demonstrated a strong association of urinary sodium with cystine excretion [17]. 

The mechanism that determines the association of increased urinary cystine excretion with increased sodium intake in cystinuric patients is unknown, as the reabsorption of cystine in the proximal tubule is sodium-independent [17,36,37]. It was speculated that high sodium intake would increase the intracellular neutral amino acid and sodium load, which might slow the apical reabsorption of cystine [11,36,37]. The present study, with normal subjects, was not able to confirm the previous findings, and thus rejected the hypothesis of an effect of dietary sodium intake, as judged by urinary excretion, on urinary cystine excretion. Although no association between dietary sodium intake and urinary cystine excretion was observed in the present study, restricting dietary sodium to 2300 mg/day (100 mmol/day) or 6 g/day of sodium chloride is recommended as part of a healthy diet, even if its potential benefit in preventing cystine stones is not supported by clinical trials [11]. Strict dietary sodium restriction to below 2 g per day is not necessary, and is difficult to achieve and maintain. 

Urine alkalinization is an important measure to increase solubility of cystine in urine. The upper limit of solubility of cystine at a physiological urine pH range between 5 and 7 is approximately 300–400 mg/L (1.3–1.7 mmol/L), but cystine solubility increases markedly when urine pH is raised to above 7.5 [26]. Urine alkalinization to reach the therapeutic target of pH 7.5 to 8.0 is a cornerstone of treatment of all patients with cystinuria and is recommended as a basic measure [10,11]. However, large doses of alkalizing agents are required to achieve the recommended urine pH range and gastrointestinal side effects can limit compliance with treatment [38,39,40]. Therefore, it is recommended to support pharmacological therapy for urine alkalinization with dietary measures.

A high-protein diet that is not compensated by higher alkali ingestion from fruits and vegetables increases the endogenous acid load [13]. Dietary intake of high amounts of the sulfur-containing amino acid methionine may reduce urine pH and counteract alkalinization therapy [13,14]. In the present study, the comparatively low urine pH and high urinary phosphate and sulfate excretion with the Western-type diet were mainly attributed to the acidifying effect of phosphoproteins and the sulfur-containing amino acids methionine and cystine, which are present in a higher proportion in animal protein sources than in foods derived from plants [14]. The high endogenous acid load with the WD was associated with a low urine pH, which could promote cystine stone formation. Reducing total protein intake on the MD and VD decreased hydrogen ion excretion and significantly increased urine pH, with concomitant reduction in urinary ammonium excretion. A vegetarian diet is particularly suitable for the dietary treatment of cystine stone disease, since the alkaline load from fruits and vegetables should reduce the amount of alkalizing agents required to achieve urine alkalinization at pH greater than 7.5.

A high urine volume is the most important factor in reducing urinary cystine concentration and preventing cystine stone formation. To maintain cystine concentration below the solubility limit of 1.3 mmol/L (300 mg/L) at pH 6 in adult patients with cystinuria, urine volume of at least 3.0 L per 24 h should be achieved [9,11,25,41,42,43,44]. To ensure adequate hydration, a urine specific gravity of less than 1.005 has been recommended [11,45]. Hydration has been found to prevent recurrent stone formation in as many as two-thirds of patients with cystinuria [46]. In a previous study of 27 adult patients with cystinuria, maintaining a daily urine volume of more than 3.0 L for a mean of 11.6 years was associated with a significant reduction in new stone formation [41]. While consuming the Western-type diet, 24-h urine volume was far below the therapeutic recommendations for cystine stone patients. Due to the higher fluid intake, mean urine volume was significantly higher on the MD (2.30 L/24 h) and the VD (2.45 L/24 h), which was also documented by urine density. The higher urine volume resulted in significantly lower urinary cystine concentration. To incorporate the effect of both urine pH and cystine concentration on the solubility of cystine, the ion activity product of cystine was estimated [25]. The ion activity product was lower with intake of the MD and VD, and thus reflected urinary cystine concentration rather than urine pH. To achieve the additional increase in urine output by approximately 1.0 L/24 h and to support continuous alkalinization in cystinuria, the consumption of beverages such as bicarbonate-rich mineral water and citrus juices is beneficial in the dietary management of patients with cystine nephrolithiasis [47]. 

A limitation of our study is the relatively small number of participants. As the study was conducted under strictly controlled conditions, a steady state of metabolism can be assumed. A prospective study of patients with cystinuria is warranted to confirm the effects observed on different diets and to evaluate the efficacy of a vegetarian diet in terms of stone activity.

## 5. Conclusions

To our knowledge, this is the first study to evaluate the impact of different diets on the urinary excretion of cystine and cysteine in healthy subjects. The present findings suggest that dietary phosphorus intake, as estimated from urinary phosphate excretion, is a predictor of urinary cystine excretion, while urinary sulfate, a biomarker for the intake of the sulfur-containing amino acids methionine and cystine, is the major determinant of urinary cysteine excretion. Neither cystine nor cysteine excretion was affected by dietary sodium intake, as judged by urinary sodium excretion. A vegetarian diet is particularly suitable for the dietary treatment of cystine stone disease, since the alkaline load from fruits and vegetables may reduce the amount of alkalizing agents required to achieve urine alkalinization at pH greater than 7.5. Further research is needed to evaluate the effects of different diets on the urinary risk profile and the progression of stone formation as outcome measures in patients with cystinuria.

## Figures and Tables

**Table 1:** Composition of the standardized Western-type, balanced mixed, and lacto-ovo-vegetarian diet.

	Western-Type Diet	Mixed Diet	Vegetarian Diet
Energy (kcal/day/MJ/day)	3590/15.01	2544/10.63	2600/10.88
Total protein (g/day)	95	65	65
Animal protein (g/day)	56	37	28
Plant protein (g/day)	39	28	37
Protein (g/kg BW/day)	1.2	0.8	0.8
Carbohydrates (g/day)	380	370	386
Fat (g/day)	132	82	84
Methionine (mg/day)	2290	1445	1122
Cystine (mg/day)	1543	918	714
Phosphorus (mg/day)	1840	1283	1457
Calcium (mg/day)	876	768	787
Potassium (mg/day)	3560	3314	6584
Sodium (mg/day) ^1^	4044	3351	2339
Fluid (mL/day)	1500	2500	2500

Abbreviations: BW, body weight; MJ, megajoules. ^1^ Calculated from urinary excretion.

**Table 2:** Urinary parameters on the standardized Western-type, balanced mixed, and lacto-ovo-vegetarian diet (*n* = 10).

	Western-Type Diet	Mixed Diet	Vegetarian Diet	WD vs. MD	WD vs. VD	MD vs. VD	Friedman Test
	Mean ± SD	Mean ± SD	Mean ± SD	*p* Value	*p* Value	*p* Value	*p* Value
Volume (L/24 h)	1.48 ± 0.38	2.30 ± 0.39	2.45 ± 0.76	0.002	0.006	0.557	0.001
Density (g/cm^3^)	1.015 ± 0.005	1.007 ± 0.002	1.007 ± 0.003	0.002	0.002	0.254	<0.001
pH	5.95 ± 0.34	6.51 ± 0.22	6.80 ± 0.21	0.002	0.002	0.004	<0.001
Cystine (µmol/24 h)	130.9 ± 35.0	96.8 ± 33.2	102.5 ± 56.3	0.131	0.064	0.695	0.046
Cystine (µmol/L)	96.4 ± 44.5	43.6 ± 18.0	46.1 ± 28.2	0.004	0.002	0.695	<0.001
Cysteine (µmol/24 h)	72.5 ± 25.5	66.8 ± 39.2	61.5 ± 43.3	0.105	0.084	0.131	0.006
Cystine-to-cysteine-ratio	1.90 ± 0.55	1.94 ± 1.11	2.07 ± 0.98	0.922	1.000	0.922	0.974
Sodium (mmol/24 h)	176 ± 40	146 ± 13	102 ± 48	0.020	0.006	0.014	0.001
Chloride (mmol/24 h)	169 ± 39	139 ± 17	106 ± 43	0.049	0.010	0.020	0.046
Potassium (mmol/24 h)	60 ± 11	77 ± 16	110 ± 20	0.006	0.002	0.002	<0.001
Calcium (mmol/24 h)	4.48 ± 1.22	3.09 ± 1.39	2.46 ± 1.33	0.002	0.002	0.004	<0.001
Ammonium (mmol/24 h)	38.7 ± 5.1	25.6 ± 6.4	18.7 ± 5.9	0.002	0.002	0.002	<0.001
Phosphate (mmol/24 h)	35.4 ± 5.5	29.4 ± 3.5	25.2 ± 6.3	0.027	0.004	0.131	0.006
Sulfate (mmol/24 h)	22.5 ± 3.1	18.1 ± 1.9	16.1 ± 3.7	0.002	0.002	0.264	0.002
Creatinine (mmol/24 h)	16.88 ± 1.97	15.32 ± 1.31	13.77 ± 3.16	0.020	0.064	0.105	0.135
AP Cystine (× 10^−22^)	3.89 ± 2.01	1.42 ± 0.73	1.27 ± 0.94	0.002	0.002	0.557	<0.001

Abbreviations: AP, ion activity product; WD, Western-type diet; MD, balanced mixed diet; VD, balanced lacto-ovo-vegetarian diet; SD, standard deviation.

**Table 3:** Univariate associations of different diets and 24 h urinary parameters with urinary cystine excretion.

Cystine	Coefficient B	95% CI	*p* Value
Diet WD	(reference)		
Diet MD	−13.91	−50.33–22.52	0.454
Diet VD	9.40	−35.65–54.46	0.682
Sodium (70 mmol/24 h)	0.04	−0.41–0.49	0.853
Sulfate (6 mmol/24 h)	−2.13	−8.48–4.22	0.510
Phosphate (10 mmol/24 h)	4.77	1.71–7.83	0.002

Abbreviations: WD, Western-type diet; MD, balanced mixed diet; VD, balanced lacto-ovo-vegetarian diet; CI, confidence interval. The *p* values are from generalized models with a per-person random effect. The Coefficient B is the estimated difference in urinary cystine excretion per difference in univariate predictor.

**Table 4:** Univariate associations of different diets and 24 h urinary parameters with urinary cysteine excretion.

Cysteine	Coefficient B	95% CI	*p* Value
Diet WD	(reference)		
Diet MD	21.98	−9.10–53.08	0.166
Diet VD	32.17	−6.29–70.64	0.101
Sodium (70 mmol/24 h)	0.22	−0.27–0.49	0.569
Sulfate (6 mmol/24 h)	5.90	0.48–11.32	0.033
Phosphate (10 mmol/24 h)	−0.31	−2.92–2.30	0.817

Abbreviations: WD, Western-type diet; MD, balanced mixed diet; VD, balanced lacto-ovo-vegetarian diet; CI, confidence interval. The *p* values are from generalized models with a per-person random effect. The Coefficient B is the estimated difference in urinary cysteine excretion per difference in univariate predictor.

## Data Availability

Data is available on request.
